# Determinants of the Continuum of Maternal Healthcare Services in Northwest Ethiopia: Findings from the Primary Health Care Project

**DOI:** 10.1155/2020/4318197

**Published:** 2020-08-26

**Authors:** Asmamaw Atnafu, Adane Kebede, Bisrat Misganaw, Destaw Fetene Teshome, Gashaw Andargie Biks, Getu Debalkie Demissie, Haileab Fekadu Wolde, Kassahun Alemu Gelaye, Mezgebu Yitayal, Tadesse Awoke Ayele, Telake Azale, Terefe Derso, Tsegaye Gebremedhin, Endalkachew Dellie

**Affiliations:** ^1^Department of Health Systems and Policy, Institute of Public Health, College of Medicine and Health Sciences, University of Gondar, Gondar, Ethiopia; ^2^Dabat Health and Demographic Surveillance System Research Centre, Institute of Public Health College of Medicine and Health Sciences, University of Gondar, Gondar, Ethiopia; ^3^Department of Epidemiology and Biostatistics, Institute of Public Health, College of Medicine and Health Sciences, University of Gondar, Gondar, Ethiopia; ^4^Department of Health Promotion and Behavioral Science, Institute of Public Health, College of Medicine and Health Sciences, University of Gondar, Gondar, Ethiopia; ^5^Department of Human Nutrition, Institute of Public Health, College of Medicine and Health Sciences, University of Gondar, Gondar, Ethiopia

## Abstract

**Background:**

The maternity continuum of care is the continuity of maternal healthcare services that a woman uses, which includes antenatal care (ANC 4+), skill birth attendant (SBA), and postnatal care (PNC) within 48 hours of delivery. It is one of the essential strategies for reducing maternal and newborn mortality. This study aimed to assess the factors associated with the completion of a continuum of maternal healthcare services among mothers who gave birth in the past five years.

**Methods:**

A community-based cross-sectional study was conducted from May 01 to June 29, 2019, among 565 randomly selected mothers who gave birth in five years before the study in primary healthcare project implementation districts of north Gondar zone, Amhara National Regional State, Ethiopia. Bivariable and multivariable logistic regression analysis were computed, and in the multivariable logistic regression analysis, adjusted odds ratio (AOR) with 95% confidence interval (CI) and a *p* value of less than 0.05 were used to identify the associated factors with completion of the continuum of maternal healthcare services.

**Results:**

The study revealed that the overall completion of the continuum of maternal healthcare services was 21.60% (95% CI: 18.20, 24.90). Women who were able to read and write (AOR: 2.70, 95% CI: 1.22, 6.04), using car/motorcycle as a means of transportation to get the health facility (AOR: 5.59, 95% CI: 2.29, 9.50), travel time less than an hour to get the health facility (AOR: 4.98, 95% CI: 2.97, 8.38), being satisfied with the service delivery (AOR: 1.89, 95% CI: 1.15, 3.11), and getting health education on maternal healthcare services in the last 6 months (AOR: 2.77, 95% CI: 1.52, 5.05) were factors associated with the completion of the continuum of maternal healthcare services.

**Conclusions:**

The completion of the continuum of maternal healthcare services was relatively low, indicating that women were not getting the likely health benefit from the present health services. Therefore, interventions should focus on increasing women's awareness, improving the availability of services at nearby health facilities, and improving service delivery by considering women's preferences and needs to increase their satisfaction are essential to increase the completion of maternal healthcare services.

## 1. Background

Maternal mortality remains an impending global health problem, with an estimated 810 deaths per day in 2017, where the vast majority of these deaths (94%) occurred in low-resource settings [1]. Sub-Saharan Africa alone accounted for approximately 67% of the maternal deaths. The maternal mortality ratio (MMR) in low-income countries is 462 per 100,000 live births and 11 per 100,000 live births in high-income countries in 2017 [2]. Similarly, Ethiopia is currently identified among the countries in the world with the highest MMR (412 deaths per 100,000 live births) [3]. As a result, policies to ensure antenatal care (ANC) follow-ups, skilled birth attendant (SBA), and postnatal care (PNC) services are essential maternal health interventions that can significantly reduce maternal mortality [4, 5].

The maternity continuum of care concept has been emphasized to improve maternal health through integrated service delivery [6–8]. It encompasses key elements of services including antenatal care (ANC), delivery with a skilled birth attendant (SBA), and postnatal care (PNC) within 48 hours of delivery for both mothers and newborns. The continuum of care recognizes that safe childbirth is critical for improving many health outcomes of both the woman and the newborn child, and it is one of the essential strategies for reducing maternal and newborn mortality [7, 9, 10]. Moreover, a well-functioning continuum of care can increase client and provider satisfaction and maximize efficiency in resource-limited settings [11, 12].

According to studies on the issue, several factors contribute to the completion of the maternity continuum of care. Socio-demographic, socioeconomic, and cultural context [13–15], including accessibility, availability, and affordability of these services play an essential role in maternal healthcare services [8, 16–18].

Improving maternal health is one of the targets of the third Sustainable Development Goals (SDGs) adopted by the international community [19]. Completing the continuum of maternal health services can reduce most of the preventable cause of maternal and neonatal death and achieve the stated targets of the Sustainable Development Goal (SDG) [20, 21].

The concept of the continuum of maternal healthcare has become one of the critical strategies for the reduction of maternal and newborn deaths and the improvement of their health and wellbeing [21–24]. However, considerable research focused on factors influencing the utilization of antenatal care, skilled birth attendance, or postnatal care separately. Therefore, instead of looking at maternal health services individually, this study sets out to examine the three components of the continuum of care, including four or more ANC visits, delivery assisted by SBA, and PNC within 48 hours for the mother in Dabat and Gondar Zuria rural districts, Ethiopia.

## 2. Methods

### 2.1. Study Design and Settings

A community-based cross-sectional study design was conducted in Gondar Zuria and Dabat districts, Northwest Ethiopia, from May 01 to June 29, 2019, to assess the completion of the continuum of maternal care. These two districts are among the six districts of primary healthcare project implementation areas in north Gondar zone, Amhara National Regional State, Ethiopia. Gondar Zuria and Dabat districts consist of 32 and 38 kebeles (the smallest administrative unit in Ethiopia), respectively. The Dabat district has four health centers and twenty-nine health posts and has administratively divided into three urban and twenty-nine rural kebeles, and it is a residence of 155,093 population. Of these, 80,648 are females. While Gondar Zuria district has five urban and thirty-three rural kebeles with 242,892 inhabitants, 116,386 males and 126,506 females. There are 37,222 and 57,322 women in the childbearing age group (15-49) in Dabat and Gondar Zuria districts, respectively. Moreover, 15,423 and 24,113 women gave birth in Dabat and Gondar Zuria districts within the past five years prior to the study, respectively.

All mothers who gave birth in the past five years before the time of data collection in the districts were the source population. Mothers with most recent births in the last five years preceding this study from each selected kebeles in both districts were included in the study. However, mothers who gave birth in another district and came to the study area and mothers critically ill and unable to respond to the interview were excluded from the study.

### 2.2. Sample Size and Sampling Techniques

The required sample was calculated by single population proportion formula (*n* = (*Z*_*α*/2_)^2^ × *P*(1 − *P*)/(*d*)^2^) with the assumptions of the proportion of continuum of care for maternal healthcare services was 50% (to get the maximum sample), a 95% confidence level, 5% marginal error (d), and adding 5% nonresponse rate, and 1.5 design effect which gives a total sample of 583.

Initially, eight kebeles from the Dabat district and ten kebeles in the Gondar Zuria district (30% of the total kebele) were selected using the lottery method. Then, the proportional allocation was applied for each selected kebeles based on the number of the mother who gave birth for the last five years before the data collection period (information obtained from the community health information system) in each kebele. Finally, the study participants were selected using simple random sampling techniques (lottery method) from the Community Health Information System (CHIS) register. Then having the name and house number, interview of the mother was conducted home to home. For those mothers who gave birth more than once in the past five years, mothers with the most recent births fulfilling the inclusion criteria were interviewed.

### 2.3. Variables and Measurements

The dependent variable of the study was the continuum of maternal healthcare services. Whereas, the independent variables were socio-demographic variables of women (age, educational status, religion and ethnicity, husbands' education status, occupational status), maternal healthcare services (antenatal care, delivery place, and postnatal care), and physical access to maternal health services and other related services.

The outcome variable continuum of maternal healthcare services was analyzed by the proportion of women who received maternal healthcare services at the pregnancy, delivery, and postdelivery stages. They are measured following Jacobs et al., definition [25]. Accordingly, when a woman receives ANC four times or more, delivers at home or in a health facility by a trained health professional, and receives maternal PNC within 48 hours after delivery by an appropriate provider, it is considered as complete the continuum of maternal healthcare services.

### 2.4. Data Collection Tools and Procedures

Data were collected using a structured interviewer-administered questionnaire, which was initially prepared in English and translated to the local language (Amharic) by language expertise, and then translated back to English by language expertise to ensure its consistency and accuracy. Fifteen diploma nurse data collectors and three public health officer field supervisors were employed for the data collection process. One day training was provided on the techniques of interviewing, handling ethical issues, maintaining confidentiality and privacy two days before the pretest, and five days before the final data collection. The tool was pretested on 5% of the sample (29 women who gave birth in the last five years) in Koladeba district (nearby the districts) to ensure the internal validity of the study.

### 2.5. Data Management and Analysis

The data was cleaned and checked for consistency, coded, and entered into Epi-Data version 3.1 software, and exported to SPSS version 20 software for analysis. Descriptive measures were computed to summarize the socio-demographic characteristics of the participants and the completion of the continuum of maternal healthcare services. Both bivariable and multivariable logistic regression analyses were computed to determine the associated factors. Variables with a *p* value of less than 0.2 in the bivariable logistic regression analysis were entered into a multivariable logistic regression analysis to control possible confounding factors after checking model fitness. Finally, a *p* value of less than 0.05 and an adjusted odds ratio (AOR) with 95% confidence level (CI) were used to declare a statistically significant association between the outcome variable and with the completion of the continuum of maternal healthcare services.

## 3. Results

### 3.1. Sociodemographic Characteristics of Participants

A total of 565 study participants responded to the interviewer-administered questionnaire with a response rate of 96.5%. The median age with inter quartile range (IQR) of the women was 30 (25, 35) years. The majority of the women (92.9%) were married.

Regarding educational status, 65.5% of women and 56.3% of their husbands were unable to read and write. Among the total women, 94.9% were housewives and involved in agricultural work. Eighty percent of women use their feet as a means of transportation to a health facility. Nearly half of the women were dissatisfied with the time spent to get the service (50.3%) and service delivery (50.8%) (Table 1).

### 3.2. Dropouts, the Continuum of Maternal Healthcare Services, and Other Characteristics

**Figure 1:**
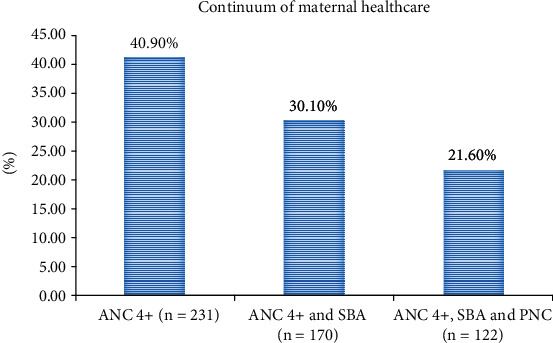
Maternal health services and the continuum of care among women who give births in the last five years, findings from the primary health care project in northwest Ethiopian districts, September 2019 (*n* = 565).

The overall continuum of maternal healthcare services among women who give recent births in the last five years preceding the study was 21.6% (95% CI: 18.2, 24.9). Nearly, forty-one percent of the women have received four and above ANC visits. However, only 30.1% of them were continued on the pathway and delivered at health facilities. After delivery, 8.5% of the women did not get the postnatal care services (Figure 1).

**Figure 2:**
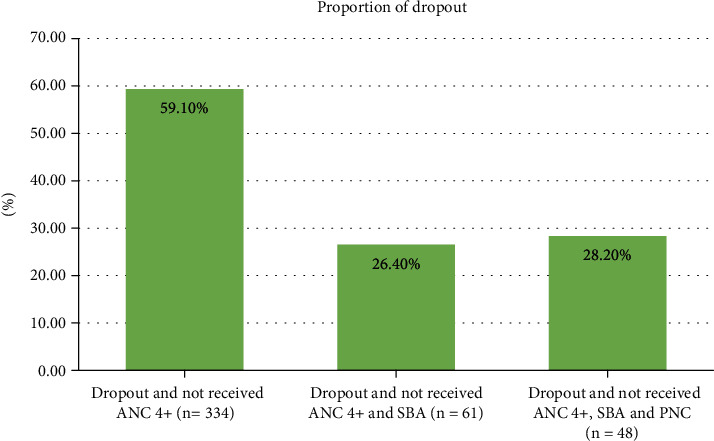
Proportion of dropout from the continuum of maternal healthcare services among women who give births in the last five years, findings from the primary health care project in northwest Ethiopian districts, September 2019 (*n* = 565).

Regarding dropout from the maternity continuum of care, a significant number of women were drop out at delivery (26.4%) and postnatal (28.2%) level (Figure 2).

### 3.3. Factors Associated with the Continuum of Maternal Healthcare Services

In the bivariable logistic regression analysis, eight variables were identified less than 0.2 significance level and included in the final multivariable logistic regression model. In the multivariable logistic regression analysis, five variables were statistically significant with the continuum of maternal healthcare services.

Accordingly, women who were able to read and write were 2.70 times more likely to complete the maternal health service continuum of care (AOR: 2.70, 95% CI: 1.22, 6.04) compared to those who were unable to read and write. Women who use cars or motorcycles for transportation were 5.59 times more likely to complete the continuum of maternal healthcare services compared to those who use their foot as a means of transportation to the health facility (AOR: 5.59, 95% CI: 2.29, 9.50). Those who were satisfied with the service delivery were 1.89 times more likely to complete the continuum of care than the dissatisfied women (AOR: 1.89, 95% CI: 1.15, 3.11). Similarly, respondents who traveled less than an hour to get the nearest health facility were 4.98 more likely to complete the continuum of maternal healthcare services than their counterparts (AOR: 4.98, 95% CI: 2.97, 8.37). Moreover, women who received health education on maternal healthcare services in the last 6 months were 2.77 times more likely to complete the continuum of maternal healthcare services than their counterparts (AOR: 2.77, 95% CI: 1.52, 5.05). (Table 2).

## 4. Discussion

This study examined the magnitude of the continuum of care for maternal healthcare services and its determinant factors among women who gave birth in the last 5 years preceding this study. Overall, 21.6% of mothers have completed the continuum of maternal healthcare services in the districts.

This result is higher than those of studies conducted in Ethiopia on completion of the continuum of maternal health care services at Arbaminch Zuria woreda, Southern Ethiopia, 9.7% [26]; EDHS 2016, 9.1% [18]; and West Gojjam Zone, 12.1% [27]. The possible explanation for the discrepancy might be due to the accessibility of health facilities, and the primary healthcare project has been implemented in the study districts in which health care services may increase utilization levels. Moreover, the majority of the women reside near the town which enables them to have access to information. The other explanation might be the use of multilevel analysis in other studies unlike that of ours.

Besides, our finding is much higher than those of studies carried out at Xaybouathong district in Lao PDR, only 6.8% continued to receive the continuum of maternal healthcare services [8], at three regions of Ghana, shows that only 8.0% of the women completed the continuum of care measured as women who received ANC4+, SBA, and PNC [17], another study finding in Ghana shows that throughout the pregnancy, delivery, and postdelivery stages, 7.9% of women and children achieved the continuum of care [28] and at four districts in Tanzania, 10% [29]. These differences could be explained by the variations in access to health and infrastructures, the study area which covers at the national level and time that might contribute to low result findings.

However, this finding is lower than the result of studies done in Nepal, in which 41% of the women received ANC, SBA, and PNC during their most recent birth [30]. Additionally, our finding is much lower than those of studies conducted at Sohag governorate, Egypt, in which 50.4% of the women had achieved continuum of care measured (ANC+4 visit, delivered by a skilled birth attendant and had PNC) [31], and a study conducted in Cambodia, 60% of women had the full range of services for the continuum of maternal and newborn health care [32]. The possible reason for this discrepancy might be the difference in sample size and socio-cultural variations. The other possible explanation could be that a longer study period retrospectively to assess the utilization that involved five years prior to the survey might increase their recall bias about the services they received for the last five years.

Our finding shows that mothers who can read and write were 2.7 more completed the continuum of care for maternal health services as compared to their counterparts. This finding is consistent with the result of studies conducted in Ghana, Egypt, Nepal, and South Asia and Sub-Saharan countries in which women's education was positively associated with the completion of the continuum of maternal healthcare services [18, 28, 31, 33]. This might be explained by the fact that education could enhance the women's knowledge, access to information, efficiency to grasp the advocacy messages through the media, and healthcare workers. Moreover, women might have more information about services provided in which maternal and child health services are exempted.

Our study identified that mothers with less than one hour of travel time to the health facility were more likely to complete the continuum of maternal health services, and also a strong association was detected between the type of transport used to health facility and completion of the continuum of care, which was congruent with the finding of other studies in which travel time [34, 35], access to healthcare, and geographical locations positively affect the completion of the continuum of care [17, 31]. This might be because women who travel to the health facility for maternal healthcare services will be less physically able to travel long distances on foot. The other possible reason might be for those who reside in the countryside might have less information and awareness about the services and socio-cultural factors that might affect their decision to get the services.

The odds of completing the continuum of maternal healthcare services were higher among participants who perceive satisfied with service delivery compared to those who were dissatisfied. This was in line with other studies [36, 37]. This might be explained by the fact that satisfied women may have positive health communications with health professionals, which makes women more motivated to continue using maternal health services.

In this study, higher odds of the continuum of care were observed among participants who get health education on maternal healthcare services in the last six months compared with their counterparts. This result was supported by other studies [15, 38]. The possible reason for this could be the health education sessions provided by health professionals to enhance women's awareness about the need for maternity care and the possible hazards of inadequate care during pregnancy; thus, it encourages the probability of looking for maternal healthcare services.

### 4.1. Contributions and Limitations of the Study

Even though this study was subjected to social desirability bias because of the administration of the interview by health professionals, we have used data collectors from other hospitals out of the districts. The other possible bias might be women might experience recall bias because of the more extended time inclusion of the study duration; particularly, they may face difficulty remembering the services they had got during their previous obstetrics visits. To overcome this, we have asked the women using rehearsal techniques and repeatedly.

## 5. Conclusion and Recommendations

In conclusion, the magnitude of the continuum of maternal healthcare services in the study area was found to be under the expected. Therefore, awareness creation for those illiterate women at the community level, fulfilling transport facilities/infrastructure, and constructing health facilities to the nearby for the majority of the residents could improve the continuum of maternal healthcare services in those reside in rural districts.

## Figures and Tables

**Table 1:** Sociodemographic characteristics of study participants' findings from the primary health care project in two northwest Ethiopian districts, September 2019 (*n* = 565).

Variable	Category	Frequency (*n*)	Percentage (%)
Age in years	15-24	97	17.2
	25-34	287	50.8
	35-49	181	32.0
Marital status	Married	525	92.9
	Unmarried	40	7.1
Mothers education	Unable to read and write	370	65.5
	Able to read and write	195	34.5
Husbands education	Unable to read and write	318	56.3
	Able to read and write	247	43.7
Mothers occupation	Housewife and agricultural work	536	94.9
	Unemployed/daily laborer	29	5.1
Means of transportation to health facility	Motorcycle/car	113	20.0
	On foot	452	80.0
Perceived required time to reach health facilities	Less or equal to 60 minutes	430	76.1
	Greater than 60 minutes	135	23.9
Know health extension worker	Yes	521	92.2
	No	44	7.8
Getting permission to go to the health facility	Big problem	80	14.2
	Not a big problem	485	85.8
Getting money to self-care	Big problem	231	40.9
	Not a big problem	334	59.1
Go alone	Big problem	295	52.2
	Not a big problem	270	47.8
Family size	<=5	281	49.7
	>5	284	50.3
Children ever born	<=3	282	49.9
	>3	283	50.1
Level of satisfaction with time spent to get the service	Dissatisfied	284	50.3
	Satisfied	281	49.7
Level of satisfaction with service delivery	Dissatisfied	287	50.8
	Satisfied	278	49.2
Level of satisfaction with the distance of the service	Dissatisfied	316	55.9
	Satisfied	249	44.1

**Table 2:** Bivariable and multivariable logistic regression analysis of continuum of maternal healthcare services among women who give births in the last five years, findings from the primary health care project in northwest Ethiopian districts, September 2019 (*n* = 565).

Variables	Category	Continuum of care		Odds ratio	
		Yes	No	COR (95% CI)	AOR (95% CI)
Women's education	Unable to read and write	114	334	1	1
	Able to read and write	8	109	4.65 (2.20, 9.83)	2.7(1.22, 6.04)^∗^
Husband's education	Unable to read and write	110	328	1	1
	Able to read and write	12	115	3.21 (1.71, 6.05)	1.86 (0.86, 3.85)
Means of transportation	Motorcycle/car	68	384	5.17 (3.29, 8.11)	5.59 (2.29, 9.50)^∗^
	On foot	54	59	1	1
Travel time to health center	Less or equal to 60 minutes	60	370	5.24 (3.39, 8.09)	4.98 (2.97, 8.37)^∗^
	Greater than 60 minutes	62	73	1	1
Perceived satisfaction with service delivery	Satisfied	42	236	2.17 (1.43, 3.29)	1.89 (1.15, 3.11)^∗^
	Dissatisfied	80	207	1	1
Getting permission to go to health facility	Not big problem	30	50	1	1
	Big problem	92	393	2.56 (1.54, 4.25)	1.82 (0.99, 3.33)
Do you know your HEW	Yes	118	403	0.34 (0.12, 0.97)	0.92 (0.26, 3.16)
	No	4	40	1	1
Receive health education on maternal healthcare services in the last 6 months	Yes	297	104	2.84 (1.65, 4.86)	2.77 (1.52, 5.05)^∗^
	No	146	18	1	1

^∗^ Statistically significant at a *p* value of less than 0.05.

## Data Availability

The data for the findings of this study are available upon request from the corresponding author.

## Abbreviations

AOR: Adjusted odds ratio
ANC: Antenatal care
CHIS: Community health information system
COR: Crude odds ratio
HEWs: Health extension workers
MMR: Maternal mortality ratio
PNC: Postnatal care
SBA: Skilled birth attendant
SDG: Sustainable Development Goal
WHO: World Health Organization.
